# Denture-Related Irritation Fibroma With Myxoid Change of the Anterior Hard Palate: A Clinicopathologic Case Report

**DOI:** 10.7759/cureus.105604

**Published:** 2026-03-21

**Authors:** Yoshiki Sato, Tetsuro Morikawa, Yoshihiro Abiko, Hiroki Nagayasu, Tsuyoshi Shimo

**Affiliations:** 1 Division of Reconstructive Surgery for Oral and Maxillofacial Region, Department of Human Biology and Pathophysiology, School of Dentistry, Health Sciences University of Hokkaido, Hokkaido, JPN; 2 Division of Oral Medicine and Pathology, Department of Human Biology and Pathophysiology, School of Dentistry, Health Sciences University of Hokkaido, Hokkaido, JPN; 3 Division of Oral Maxillofacial Surgery for Oral and Maxillofacial Region, Department of Human Biology and Pathophysiology, School of Dentistry, Health Sciences University of Hokkaido, Hokkaido, JPN

**Keywords:** alcian blue, denture-related irritation fibroma, denture-related trauma, hard palate, myxoid change, oral focal mucinosis

## Abstract

Denture-related irritation fibroma is a common reactive lesion of the oral mucosa caused by chronic mechanical irritation; however, cases arising on the anterior hard palate and exhibiting prominent myxoid stromal change are rare. A 54-year-old man presented with a long-standing, pedunculated mass (16 × 20 × 11 mm) on the right anterior hard palate in the setting of long-term maxillary complete denture use. After an incisional biopsy revealed moderate epithelial dysplasia, the lesion was excised under local anesthesia with a 2 mm safety margin at the stalk and subperiosteal dissection.

Histopathology revealed a fibrous nodule covered by stratified squamous epithelium with hyperkeratosis and acanthosis, and a well-demarcated area of myxoid matrix immediately beneath the epithelium. Alcian blue (AB) staining showed diffuse stromal positivity, and immunohistochemistry demonstrated a very low Ki-67 labeling index and broad-spectrum cytokeratin (CK-wide) negativity in the lesional stroma, with diffuse epithelial positivity as an internal control, supporting a diagnosis of denture-related irritation fibroma with myxoid stromal change. Postoperative healing was uneventful, and no recurrence was observed after denture adjustment and improvement of denture hygiene.

In this report, we present a case of denture-related irritation fibroma with moderate epithelial dysplasia and prominent myxoid stromal change that had remained untreated and progressively enlarged over approximately 20 years.

## Introduction

Irritation fibroma is a common reactive hyperplastic lesion of the oral mucosa, usually arising in response to chronic mechanical irritation from teeth, restorations, or prostheses (denture-related irritation fibroma) [1]. Clinically, it presents as a well-circumscribed, sessile or pedunculated nodule with a smooth surface, most often on the buccal mucosa or tongue, whereas palatal lesions are less frequently reported. Histopathologically, conventional lesions are composed of dense collagenous stroma, with varying degrees of chronic inflammation and only limited stromal myxoid change [1]. Irritation fibroma is commonly associated with chronic local irritation, such as trauma from dentures, occlusal forces, or other mechanical stimuli. Long-standing irritation may induce stromal alterations, including myxoid degeneration.

In contrast, interstitial myxoid change, resulting from the accumulation of hyaluronic acid-rich mucinous ground substance, may be observed as an associated finding in irritation fibroma [2-4]. Because prominent stromal myxoid change is uncommon in conventional irritation fibroma, it can create a diagnostic pitfall and requires careful clinicopathologic correlation. However, when this myxoid component becomes prominent or even dominant, differentiation from oral focal mucinosis (OFM) or mucinous lesions of minor salivary gland origin, such as adenomatoid hyperplasia, can be challenging [2-4]. In such situations, careful correlation of the clinical context with routine histology and special stains, such as Alcian blue (AB), with or without prior hyaluronidase digestion, as well as immunohistochemistry for epithelial markers, is essential for an accurate diagnosis [2-4].

Irritation fibroma commonly occurs on the gingiva, buccal mucosa, palate, and lips; on the buccal mucosa, it frequently arises along the occlusal line of the maxillary and mandibular teeth; on the tongue, it tends to involve the anterior dorsal surface; and on the lips, it predominantly affects the lower lip. Although the anterior hard palate is not a common site for irritation fibroma, reactive fibrous lesions may develop in this region in the presence of chronic mechanical irritation, such as that caused by removable dentures [1,5].

We report a case of a denture-related irritation fibroma with relatively prominent myxoid stromal change, arising beneath a removable maxillary denture in a 54-year-old man, which had remained unexcised for approximately 20 years with relief of the palatal denture base, and discuss its clinicopathologic features.

## Case presentation

Patient's medical and family history

A 54-year-old man was referred to our department on August 13, 2025, with a complaint of a palatal mass. The patient’s past medical and family histories were unremarkable.

History of present illness

The patient had first noticed a palatal swelling approximately 20 years earlier, but had not sought medical attention because of his busy schedule. His maxillary complete denture had been fabricated eight years previously and had been used continuously without major adjustment. The lesion likely pre-existed the denture use, and chronic mechanical irritation from the denture may have contributed to its gradual enlargement and persistence.

Clinical findings

**Figure 1:**
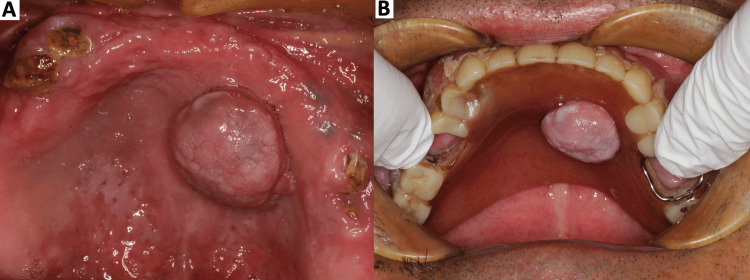
Intraoral photographs at the first visit A) After denture removal, a pedunculated, smooth-surfaced nodule measuring 16 × 20 × 11 mm is present on the right anterior hard palate; the mucosal surface is intact, without ulceration, and the lesion is freely movable on palpation. B) The denture base had been relieved and adjusted to follow the external contour of the lesion.

**Figure 2:**
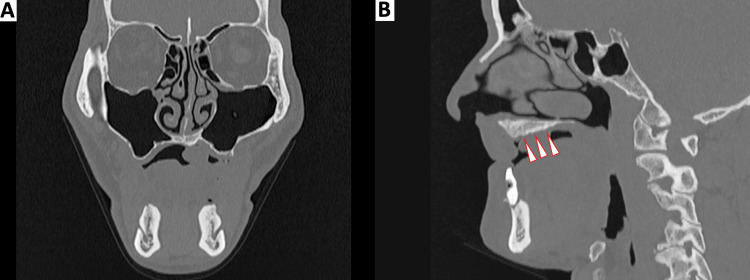
Computed tomography (CT) findings of the palatal lesion A) Coronal CT image showing a soft-tissue mass on the right anterior hard palate without cortical erosion of the palatal bone. B) Sagittal CT image confirming the absence of underlying bony resorption beneath the lesion; the arrow indicates the intact palatal bone surface.

Intraoral examination revealed a pedunculated mass on the right anterior hard palate measuring 16 × 20 × 11 mm, with a smooth surface and no ulceration (Figure 1A). The lesion was asymptomatic; the patient reported no pain or bleeding, and no functional disturbance, such as dysphagia or speech difficulty, aside from awareness of the mass. The lesion was freely movable on palpation, with an elastic to slightly firm consistency, and no induration was detected. The maxillary complete denture had been fabricated with relief of the palatal base to accommodate the external contour of the lesion (Figure 1B). Helical computed tomography (CT) of the maxilla demonstrated a soft-tissue mass on the right anterior hard palate, without cortical erosion or resorption of the underlying palatal bone (Figures 2A-2B). There was no involvement of the nasal floor or maxillary sinus.

Systemic examination showed a moderately built man in good nutritional condition. Extraoral examination revealed a symmetrical facial appearance without abnormalities in the regional lymph nodes.

Clinical diagnosis

Based on the clinical findings and the presence of chronic denture irritation, a denture-related irritation fibroma was clinically suspected.

Treatment and clinical course

**Figure 3:**
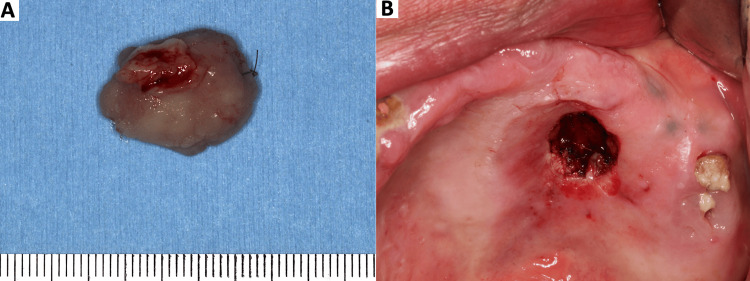
Gross photograph of the excised lesion and postoperative intraoral photograph A) Gross photograph of the excised pedunculated lesion with its stalk. B) Postoperative intraoral photograph after subperiosteal excision of the lesion.

At the initial visit, the procedure was explained to the patient, and an incisional biopsy of the lesion was performed under local anesthesia. The biopsy specimen showed a lesion covered by keratinized to parakeratinized stratified squamous epithelium, with elongation of the rete ridges. The underlying lamina propria consisted of a proliferation of fibrous connective tissue, with a mild chronic inflammatory cell infiltrate. No histologic evidence of invasive epithelial growth or stromal invasion was observed. On this basis, the biopsy was interpreted as moderate epithelial dysplasia. Based on the biopsy findings, the lesion was completely excised, including the pedunculated stalk. The gross specimen (Figure 3A) and the surgical defect immediately after excision (Figure 3B) are shown. The excised specimen was fixed in 10% neutral buffered formalin and submitted for histopathologic examination.

Postoperatively, the patient reported no pain or bleeding, and epithelialization of the surgical wound progressed slowly but steadily. Because considerable plaque and staining were observed on the denture, detailed instructions on denture hygiene were provided. One month after surgery, the patient was free of pain and able to eat without difficulty. The previously exposed palatal bone surface was completely covered by mucosa, and epithelialization of the surgical site was satisfactory. No recurrence of whitish lesions around the excision margins was observed.

The patient has since been followed up periodically, and no evidence of recurrence has been noted.

Histopathological findings

Excisional Specimen (Final Diagnosis)

**Figure 4:**
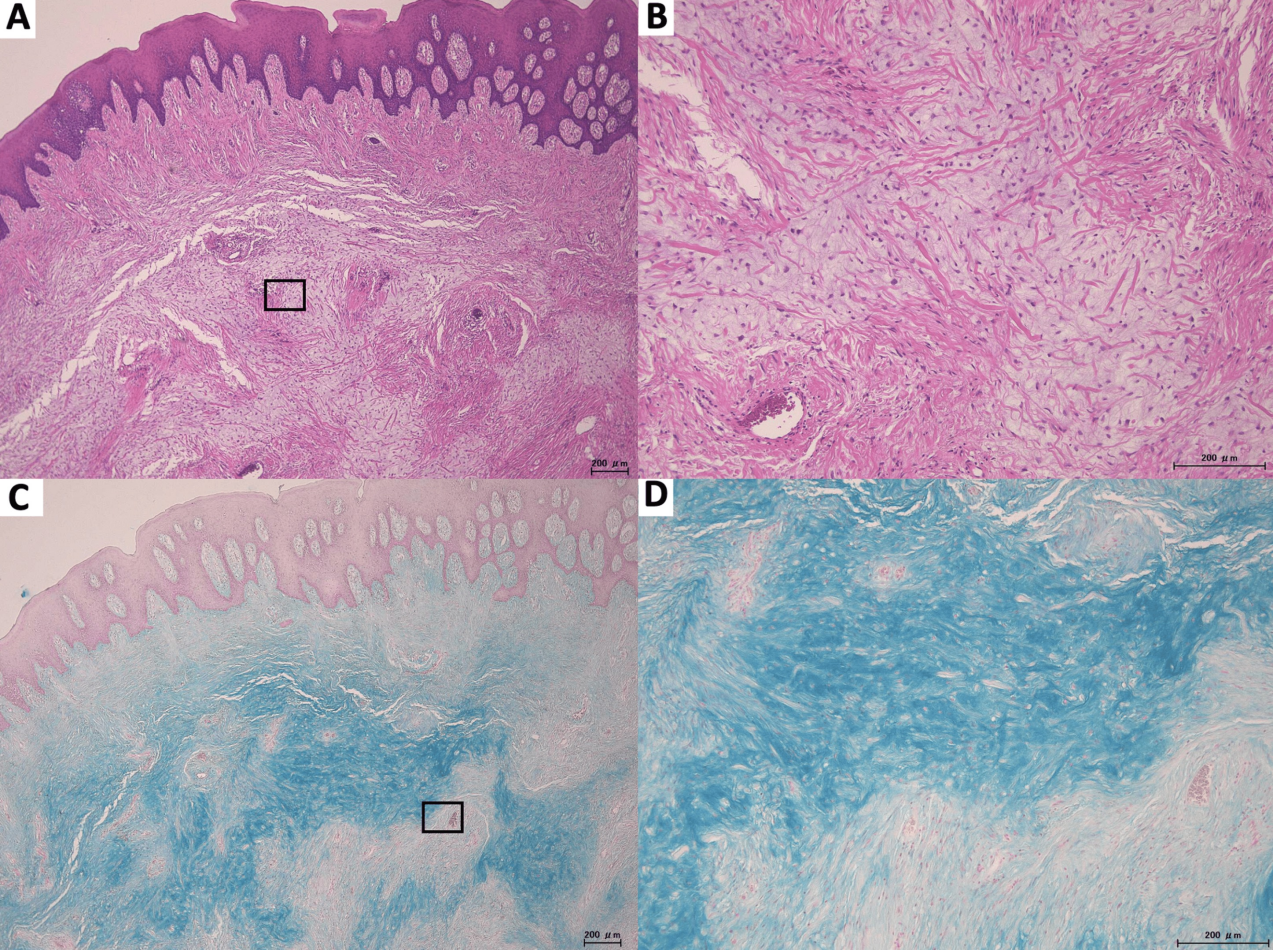
Histopathological findings of the excised lesion A) Low-power photomicrograph (H&E) showing a pedunculated irritation fibroma, lined by ortho- to parakeratinized stratified squamous epithelium with elongated rete ridges overlying a fibrous stroma. The boxed region in panel A is shown at higher magnification in panel B. B) Medium-power view (H&E), demonstrating a densely collagenous stroma with chronic inflammatory cell infiltration and prominent myxoid change in the lamina propria. C) Low-power view (Alcian blue) showing diffuse Alcian blue-positive myxoid matrix within the stromal connective tissue. The boxed region in panel C is shown at higher magnification in panel D. D) Higher-power view (Alcian blue), confirming that the Alcian blue-positive material is distributed in the interstitial stroma rather than within glandular or ductal lumina. Scale bar: 200 μm

The excisional specimen consisted of a pedunculated nodular lesion covered by orthokeratinized to parakeratinized stratified squamous epithelium, with elongated rete ridges (Figure 4A). Beneath the epithelium, the lamina propria contained dense fibrous connective tissue, with chronic inflammatory cell infiltration composed predominantly of lymphocytes and plasma cells. The stroma exhibited deposition of mucinous material (myxoid matrix) on a background of fibrous connective tissue (Figure 4B). Special staining with AB demonstrated diffuse positivity, with the positive material distributed within the interstitial stroma (Figures 4C-4D).

**Figure 5:**
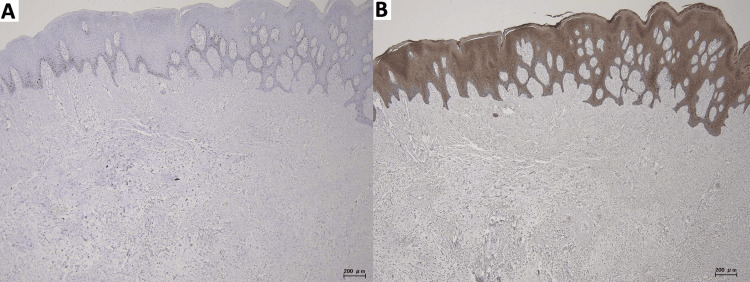
Immunohistochemical findings A) Ki-67 immunostaining shows a very low nuclear labeling index, indicating no increased proliferative activity. B) Cytokeratin wide (CK-wide) immunostaining highlights the surface epithelium (internal positive control), while the lesional stroma is negative. Scale bar: 200 μm

Immunohistochemical staining showed a very low Ki-67 labeling index (Figure 5A). The lesional stromal cells were negative for broad-spectrum cytokeratin (CK-wide), whereas the surface epithelium showed diffuse positivity as an internal positive control (Figure 5B). Taken together, these findings, in the clinical setting of chronic denture-related trauma, led to a final diagnosis of denture-related irritation fibroma with myxoid stromal change.

## Discussion

In this case, a pedunculated mass was present on the right anterior hard palate in a patient with a history of long-term denture use. Histologically, the lesion showed features of irritation fibroma, with a covering of stratified squamous epithelium and elongated rete ridges, in combination with myxoid change in the underlying stroma. Special staining revealed AB-positive material within the interstitial stroma (Figure 4).

Immunohistochemically, the stroma was negative for CK-wide. Irritation fibroma is a reactive lesion, characterized by reparative fibrous proliferation in response to chronic mechanical irritation. Long-term denture-related irritation, including poor fit and inadequate denture hygiene, is one of the major etiologic factors [1,5]. Myxoid change in irritation fibroma is considered to represent an interstitial alteration caused by excessive production of acidic mucopolysaccharides (such as hyaluronic acid) by fibroblasts, and it variably coexists with fibrous stroma, depending on the degree of chronic inflammation [1-3].

Nakamura et al. demonstrated stromal histopathologic heterogeneity in 40 cases of oral irritation fibroma and proposed a classification that included myxoid and mixed types [1]. Among these, the pure myxoid type accounted for 7 of 40 cases (17.5%), and, even when mixed-type lesions (n = 4) were included, lesions containing myxoid components comprised only 11 of 40 cases (27.5%) [1]. Thus, although myxoid stromal change is a recognized finding in irritation fibroma, a distinctly myxoid pattern appears to represent a minority histopathologic presentation [1]. The significance of the present case lies in the fact that such an irritation fibroma with myxoid change arose in the anterior hard palate, where OFM and minor salivary gland-derived lesions are clinically relevant differential diagnoses, and could be reasonably interpreted as a reactive fibrous lesion through integration of multimodal findings.

When myxoid change is observed in the stroma, differential diagnosis from OFM becomes a major consideration. Morphologically, irritation fibroma often presents as a pedunculated, polypoid lesion with predominant fibrous stroma, whereas OFM has been reported as a small lesion with a myxoid connective tissue background, in which mucin deposition is prominent and collagen fibers are reduced [4]. In the present case, the lesion was clearly pedunculated and exhibited a well-developed fibrous stroma, favoring irritation fibroma over OFM on morphologic grounds. Other benign myxoid lesions reported in the oral cavity include superficial angiomyxoma and nerve sheath myxoma [6,7]. Interpretation of special stains is also critical. AB at pH 2.5 can stain a broad spectrum of acidic mucopolysaccharides; therefore, the key determinant is not simply whether staining is present, but where it is localized. AB positivity in the interstitial stroma supports the presence of interstitial mucopolysaccharides, whereas positivity confined to glandular or cystic lumina suggests intraluminal mucin. In this case, AB-positive material was confined to the stromal compartment, and no obvious proliferation of glandular structures was identified. Furthermore, the negativity of the stromal component for CK-wide does not support a proliferation of epithelial glandular elements.

In addition, helical CT showed no resorption or destruction of the palatal bone beneath the lesion, supporting the interpretation that the process was confined to the mucosa and submucosa, rather than representing an intraosseous or aggressive lesion.

Representative cases of oral fibroma showing myxoid stromal change are summarized in Table 1 [8-10].

**Table 1:** Reported cases of irritation fibroma with myxoid stromal change Abbreviations: IHC, immunohistochemistry; NR, not reported Size indicates the clinical size unless otherwise specified.

Author	Age	Sex	Location	Size (mm)	Clinical diagnosis	Final pathological diagnosis	Stains	Recurrence
Lanjekar et al. (2016) [8]	37	F	Left mandibular alveolar ridge	50 × 40 × 30	Irritation fibroma	Irritation fibroma (myxoid areas)	H&E	NR
Gupta et al. (2017) [9]	36	F	Left mandibular alveolar ridge	60 × 50 × 30	Irritation fibroma	Irritation fibroma	H&E; IHC: Vimentin+, S-100-	No (14 mo)
Shahpaska et al. (2024) [10]	67	F	Left maxillary alveolar ridge	25 × 15 × 10	Irritation fibroma	Irritation fibroma (myxoid degeneration)	H&E	No (6 mo)
Sato et al. (present case)	54	M	Right maxillary hard palate	16 × 20 × 11	Irritation fibroma	Irritation fibroma with myxoid stromal change	H&E; Alcian blue+; IHC: CK13-/CK17-; Ki-67 low/negative; CK-wide-	NR

Differential diagnosis of minor salivary gland lesions is also important. In lesions such as adenomatoid hyperplasia or those with ductal ectasia, increased numbers of acini and ducts are typically observed histologically. In addition, intraluminal mucin often shows positivity for mucicarmine, and the epithelial component is positive for epithelial markers, such as CK7 and CK19, while myoepithelial markers, such as SMA and p63, provide supportive evidence of salivary gland origin [11,12]. In the present case, there was no clear evidence of increased glandular structures, and, when combined with the stromal negativity for CK-wide and the clinically pedunculated morphology, the involvement of minor salivary gland-derived pathology was considered unlikely.

In the biopsy specimen of this case, moderate oral epithelial dysplasia (OED) was reported. Reactive epithelial hyperplasia and dysplasia of the surface epithelium adjacent to irritation fibroma can occur under conditions of chronic irritation, but malignant transformation is uncommon [13]. From a clinical management perspective, en bloc excision with histologic assessment of surgical margins is recommended, followed by periodic visual examination and photographic documentation every three to six months, tailored to the presence or absence of residual dysplasia. In the present case, after complete excision of the lesion, bone exposure disappeared, epithelialization was satisfactory, and no signs of recurrence have been observed during follow-up.

The fundamental principles of treatment are complete excision of the lesion and elimination of causative irritants. In denture-related cases, detailed denture hygiene instruction, avoidance of nocturnal denture wearing, reduction of localized pressure, and, when necessary, denture adjustment or remaking, contribute to preventing recurrence [14]. In the present case, these interventions were effective, and the short-term prognosis has been favorable.

In summary, when the clinical features (a pedunculated lesion on the anterior hard palate associated with long-term denture-related irritation), histologic findings (a fibrous stroma-predominant lesion with interstitial myxoid change), radiographic findings (helical CT showing no resorption or destruction of the underlying palatal bone), and staining/immunohistochemical findings (stromal AB positivity distributed within the interstitial stroma, CK-wide negativity in the lesional stroma, and a very low Ki-67 labeling index) are considered together, it is most appropriate to interpret this lesion as a denture-related irritation fibroma with myxoid stromal change, with OFM included in the differential diagnosis.

## Conclusions

An irritation fibroma with myxoid stromal change, particularly when associated with chronic irritation from removable dentures, can arise on the anterior hard palate and closely mimic OFM or myxoid lesions of minor salivary gland origin, both clinically and histopathologically. Recognition of this pattern and careful clinicopathologic correlation, including assessment of the lesion site, history of chronic prosthetic irritation, and characteristic AB-positive myxoid stroma without a salivary or epithelial immunoprofile, are essential for accurate diagnosis and to avoid overdiagnosis of salivary gland pathology. Clinicians should consider irritation fibroma with myxoid change in the differential diagnosis of myxoid palatal lesions in patients with removable dentures, and manage these cases with complete surgical excision and elimination of chronic mechanical irritation through appropriate denture adjustment to minimize the risk of recurrence.
